# Exchange Bias Tuning for Magnetoresistive Sensors by Inclusion of Non-Magnetic Impurities

**DOI:** 10.3390/s16071030

**Published:** 2016-07-04

**Authors:** Parikshit Pratim Sharma, Edoardo Albisetti, Marco Monticelli, Riccardo Bertacco, Daniela Petti

**Affiliations:** Dipartimento di Fisica, Politecnico di Milano, via Giuseppe Colombo, 81, 20133 Milano, Italy; parikshitpratim.sharma@polimi.it (P.P.S.); marco.monticelli@polimi.it (M.M.); riccardo.bertacco@polimi.it (R.B.)

**Keywords:** magnetic tunneling junction, magnetoresistive sensors, exchange bias, IrMn, blocking temperature, field cooling

## Abstract

The fine control of the exchange coupling strength and blocking temperature ofexchange bias systems is an important requirement for the development of magnetoresistive sensors with two pinned electrodes. In this paper, we successfully tune these parameters in top- and bottom-pinned systems, comprising 5 nm thick Co_40_Fe_40_B_20_ and 6.5 nm thick Ir_22_Mn_78_ films. By inserting Ru impurities at different concentrations in the Ir_22_Mn_78_ layer, blocking temperatures ranging from 220 °C to 100 °C and exchange bias fields from 200 Oe to 60 Oe are obtained. This method is then applied to the fabrication of sensors based on magnetic tunneling junctions consisting of a pinned synthetic antiferromagnet reference layer and a top-pinned sensing layer. This work paves the way towards the development of new sensors with finely tuned magnetic anisotropies.

## 1. Introduction

Magnetic sensors have been applied to different fields [1,2], ranging from automotive applications [3,4] to biology [5]. In particular, magnetoresistive biosensors have been widely investigated for their high sensitivity, portability and the lack of magnetic background in most biological samples. Magnetoresistive sensors in combination with magnetic nanoparticles have been employed for detecting synthetic [6,7,8] and natural DNA and proteins [9]. In all these applications, the sensitivity of the experiment is determined not only by the sensor performance, but also by different factors such as the selectivity and specificity of the biological probes, the methods employed for sample preparation, the binding of the target in the biological sample, the labelling and the detection scheme [6,10].

For some challenging applications, such as the detection of neural magnetic fields, the extremely weak signals require one to push further the sensor detection limits. Spin valve (SV) and MgO-barrier magnetic tunneling junctions (MTJs) have been intensively investigated for this purpose [11,12,13]. In both these structures, the low-field sensitivity and signal-to-noise ratio depend on the magnetic configuration of the electrodes, in particular on the switching behavior of the “free layer” and on the alignment of the magnetizations in the reference and sensing layers. For achieving a linear and hysteresis-free response to external magnetic fields, the magnetic anisotropy of the sensor “free layer” should be perpendicular to that of the pinned one. When applying a magnetic field along the hard axis of the free layer, its magnetization rotates coherently, giving rise to a linear magnetoresistance curve. To obtain such a configuration, one electrode, the reference layer, is usually pinned through a synthetic antiferromagnet (SAF) structure [14], while the anisotropy of the free (sensing) layer is controlled through shape anisotropy [15] or through a magnetic biasing field. However, these solutions require, in most cases, a complex device design, often with the introduction of permanent magnets [16,17] or the application of external fields [18]. Another strategy, typically employed in MTJs, is the growth of a free layer with a superparamagnetic behavior or displaying perpendicular magnetic anisotropy (PMA) [15,19,20], resulting in a hysteresis free, linear magnetization vs. external magnetic field (M vs. H) curve. However, the low thickness required to achieve such magnetic configurations limits the magnetoresistance values [21].

More recently, a configuration with two pinned electrodes with crossed anisotropies has been implemented in MTJs [22,23,24]. This configuration is achieved through exchange bias [25], performing two subsequent field coolings along orthogonal directions at different temperatures. In this case, the anisotropy of the sensing electrode is determined by the exchange coupling strength; therefore, a tuning of the exchange bias allows to modulate the dynamic range and sensitivity of the sensor. This approach allows to tune the sensor response avoiding the problems related to the reduction of the free layer thickness and to achieve crossed configuration without introducing external biasing fields or shape anisotropy.

Concerning the annealing treatments, a key parameter, which defines the annealing temperature, is the blocking temperature (T_B_) of the system, defined as the temperature at which the exchange bias vanishes. Commonly, in the prototypal IrMn-based system, the tuning of the blocking temperature is achieved by controlling the thickness of the antiferromagnet (AFM) [26,27]. In most cases, the reference layer thickness is large enough to ensure good thermal stability and a blocking temperature above 250 °C, while the sensing layer is thinner, thus displaying a lower blocking temperature of about 200 °C [23,27]. However, it is not straightforward, by only tuning the AFM thickness, to achieve a fine control over T_B_ while preserving both a high exchange bias value and a good thermal stability of the interface [28]. On the other hand, if the two blocking temperatures are too close, the second annealing influences the reference SAF structure [22,24], or does not cross the blocking temperature of the sensing layer [24], in both cases giving rise to a not perfectly orthogonal magnetic configuration of the electrodes. This may in turn give rise to non-linearities and hysteresis in the magnetoresisitve R(H) curve, which may affect the ultimate performance of the sensor in terms of sensitivity and accuracy.

In this paper, we present a straightforward method to tune the blocking temperature and the exchange coupling strength in the top-pinned Co_40_Fe_40_B_20_/Ir_22_Mn_78_ and bottom-pinned Ir_22_Mn_78_/Co_40_Fe_40_B_20_ systems by introducing Ru impurities in the Ir_22_Mn_78_ film. By using different Ru concentrations, we modulate the blocking temperature in the 220 °C–100 °C range and the exchange bias field in the range 200 Oe–60 Oe. Differently from other works [22], our structure does not include an interlayer between Co_40_Fe_40_B_20_ and Ir_22_Mn_78_, and exchange bias values above 50 Oe are shown also in the samples with the lowest blocking temperature. This method can be successfully employed to create different exchange bias structures with a fine control of their blocking temperature and exchange coupling strength. This feature is important both for the development of magnetic sensor and thermally assisted magnetic random access memory (TA-MRAM) devices [29], but also for the fabrication of sample with tunable magnetic anisotropies [30] and domain wall configurations [31,32,33].

In this context, we applied this method to grow a magnetic tunneling junction based sensor constituted by a pinned SAF as reference layer and a top-pinned Co_40_Fe_40_B_20_ film with a blocking temperature of 140 °C and an exchange bias of 70 Oe as sensing layer. In this structure, with two subsequent field coolings along perpendicular directions, we successfully set a crossed anisotropy configuration, reaching tunneling magnetoresistance (TMR) values above 60%. Despite the introduction of an additional layer and the need for two subsequent annealing steps, the double exchange bias structure represents a viable route for combining high TMR ratio and wide tunability of the characteristics in MTJ-based sensors.

## 2. Materials and Methods

### 2.1. Sample Fabrication

The samples were deposited on Si/SiO_2_ (1000 nm) substrates by DC and RF magnetron sputtering using an Orion8 system (AJA, Scituate, MA, USA) with a base pressure of 5 × 10^−9^ Torr. The parameters used are listed in Table 1. During the deposition, a 300 Oe magnetic field (H_G_) was applied in the sample plane for setting the magnetocrystalline uniaxial anisotropy direction in the Co_40_Fe_40_B_20_ layer and the exchange bias direction in the as-grown sample.

For the study of the tuning of the exchange bias field and blocking temperature, samples with different concentration of Ru impurities were grown, keeping constant the number of [Ir_22_Mn_78_/Ru] repetitions and varying the thickness of the Ru layer from 0.05 nm to 0.14 nm. It is worth noticing that the Ru deposition does not give rise to a continuous layer, and therefore the “thickness” notation should be considered as an indication of the amount of Ru intercalated in the IrMn layer. Two configurations were studied, namely top-pinned (series A) and bottom-pinned (series B):

- Sample A0 0% Ru: Co_40_Fe_40_B_20_(5 nm)/Ir_22_Mn_78_(6.5 nm)/Ru(2 nm)
- Sample A1 8.5% Ru: Co_40_Fe_40_B_20_(5 nm)/[Ir_22_Mn_78_(0.5 nm)/Ru(0.05 nm)]_12_/Ir_22_Mn_78_(0.5 nm)/Ru(2 nm)
- Sample A2 17.5% Ru: Co_40_Fe_40_B_20_(5 nm)/[Ir_22_Mn_78_(0.5 nm)/Ru(0.09 nm)]_12_/Ir_22_Mn_78_(0.5 nm)/Ru(2 nm)
- Sample A3 21% Ru: Co_40_Fe_40_B_20_(5 nm)/[Ir_22_Mn_78_(0.5 nm)/Ru(0.11 nm)]_12_/Ir_22_Mn_78_(0.5 nm)/Ru(2 nm)
- Sample A5 Ru interlayer: Co_40_Fe_40_B_20_(5 nm)/Ru(0.2 nm)/Ir_22_Mn_78_(6.5 nm)/Ru(2 nm)
- Sample B0 0% Ru: Ir_22_Mn_78_(6.5 nm)/Co_40_Fe_40_B_20_ (5 nm)/Ru(2 nm)
- Sample B2 17.5% Ru: [Ir_22_Mn_78_(0.5 nm)/Ru(0.09 nm)]_12_/Ir_22_Mn_78_(0.5 nm)/Co_40_Fe_40_B_20_(5 nm)/Ru(2 nm)
- Sample B4 25% Ru: [Ir_22_Mn_78_(0.5 nm)/Ru(0.14 nm)]_12_/Ir_22_Mn_78_(0.5 nm)/Co_40_Fe_40_B_20_(5 nm)/Ru(2 nm)

The as grown samples underwent an annealing at 200 °C for 5 min which promoted the crystallization of the amorphous CoFeB and the IrMn layers [34,35]. Subsequently, a field cooling was performed with a 4 kOe magnetic field applied in the same direction of H_G_. The square hysteresis loops measured along this direction after the thermal treatment reveals that the procedure was effective in setting the unidirectional anisotropy of the system.

**Figure 1:**
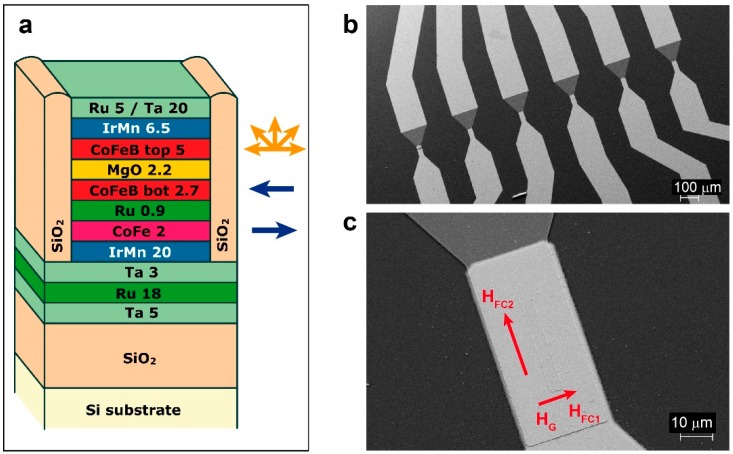
(**a**) Sketch of the sensor stack deposited by magnetron sputtering; (**b**) SEM image of the chip layout after optical lithography and ion milling; (**c**) SEM image of a single sensor. The red arrows mark the direction of the magnetic field applied during the first (H_FC1_) and second (H_FC2_) field cooling.

MTJ-based sensors with two pinned Co_40_Fe_40_B_20_ layers were grown on a Si/SiO_2_ substrate by magnetron sputtering in the same AJA Orion8 system and with the same applied magnetic field H_G_ of 300 Oe [36,37]. The stack composition was the following: Ta(5 nm)/Ru(18 nm)/Ta(3 nm)/Ir_22_Mn_78_(20 nm)/Co_60_Fe_40_(1.8 nm)/Ru(0.9 nm)/Co_40_Fe_40_B_20_(2.7 nm)/MgO(2.4 nm)/Co_40_Fe_40_B_20_(5 nm)/[Ir_22_Mn_78_(0.5 nm)/Ru(0.09 nm)]_12_/Ir_22_Mn_78_(0.5 nm)/Ru(5 nm)/Ta(20 nm). Co_60_Fe_40_ and MgO layers were deposited in RF mode while the remaining layers were grown in DC mode. Subsequently, the samples were processed with optical lithography and ion beam etching in order to obtain arrays of 3 × 40 µm^2^ magnetic tunneling junctions, with the shorter side parallel to the easy-axis of the bottom pinned layer (see the scanning electron microscope image of Figure 1b) [38]. A 100 nm thick SiO_2_ layer was deposited for insulating purposes. Afterwards, Ti (7 nm)/Au (150 nm) contact layers were deposited by magnetron sputtering. The sensors arrays were then annealed at a temperature T_FC1_ = 290 °C in vacuum (10^−6^ Torr) for 1 hour and subsequently field-cooled in a 4 kOe magnetic field (H_FC1_ in Figure 1c) applied in the direction of H_G_ (see Figure 1c). This step was performed to enable the crystallization of the ferromagnetic (FM) layers and of the MgO barrier, thus promoting coherent tunneling and to pin both the bottom and top Co_40_Fe_40_B_20_ layers in the same direction. The second annealing step was performed at a temperature T_FC2_ < T_FC1_, i.e., at 150 °C, and followed by a field cooling with the magnetic field (H_FC2_ = 4000 Oe in Figure 1c) applied perpendicular to H_G_. This step set the magnetic anisotropy direction in the top layer perpendicular with respect to the bottom one.

### 2.2. Sample Characterization

Imaging of the sensors was performed with a scanning electron microscope (SEM; Leo 1525 Raith, Zeiss-Raith, Jena-Dortmund, Germany). The hysteresis loop of the films was measured with a vibrating sample magnetometer (VSM; EZ-9, Microsense, Lowell, MA, USA). The blocking temperature of the system, defined as the temperature at which the exchange bias vanishes, was measured performing hysteresis loops at different temperatures. Magnetoresistance characterization was carried out by means of two-probes measurements at room temperature. The external magnetic field was generated by a custom electromagnet powered by a stabilized bipolar current generator (Kepco, New York, NY, USA).

## 3. Results

### 3.1. Blocking Temperature and Exchange Bias Field in Top- and Bottom-Pinned Samples

**Figure 2:**
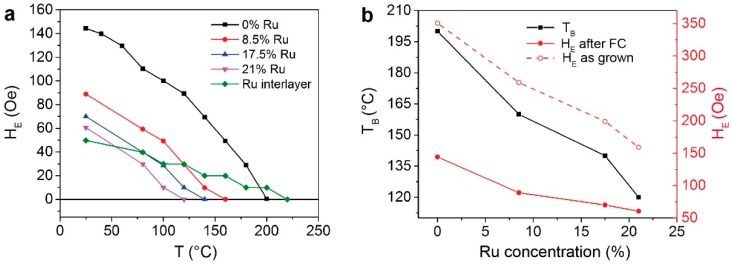
(**a**) Exchange bias field as a function of temperature at different Ru concentrations on top-pinned samples (A series); (**b**) Effect of different Ru concentrations on the blocking temperature and exchange bias field for top-pinned samples.

**Figure 3:**
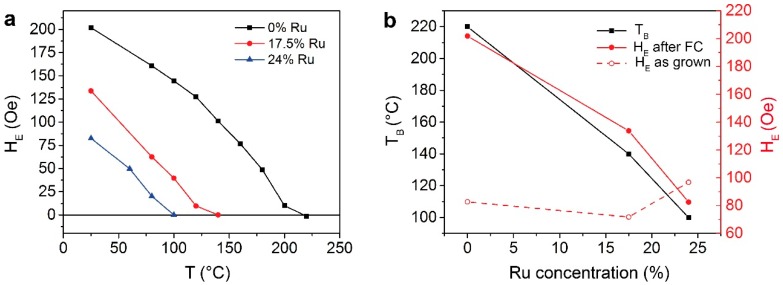
(**a**) Exchange bias field as a function of temperature at different Ru concentrations on bottom-pinned samples (B series); (**b**) Effect of different Ru concentrations on the blocking temperature and exchange bias field for bottom-pinned samples.

Figure 2a shows the exchange bias field (H_E_) as a function of the temperature for different concentrations of Ru for type A series, after the annealing at 200 °C (see Materials and Methods). As expected, in all cases the exchange bias field diminishes with the temperature, due to the fact that the fraction of IrMn grains contributing to the pinning of the CoFeB layer decreases with temperature. Indeed, in polycrystalline films like sputtered IrMn, the distribution of the grain sizes leads to a distribution in the blocking temperatures of the system [27]. The blocking temperature (T_B_) of the whole system corresponds to the temperature at which the exchange bias field goes to zero, i.e., at which the hysteresis loop is centered, and is reported in panel b of Figure 2 as a function of the Ru concentration (solid black line). As one can observe in Figure 2a,b, T_B_ decreases from 200 °C to 120 °C when the Ru concentration is increased from 0% to 21%. The same consideration applies to the exchange bias value both in the as grown sample, dashed red line in Figure 2b, and after the first annealing, solid red line in Figure 2b. In particular, exchange bias field ranging from 150 to 60 Oe are obtained after the first field cooling for Ru concentrations ranging from 0% to 21%. As it can be observed in Figure 2b and Figure 3b, in both A and B series, the as-grown samples present exchange bias along H_G_ [39]. Note, however, that in the case of top-pinned CoFeB (Figure 2), the exchange bias field decreases upon annealing. This reduction has already been observed in spin-valve structures with top-pinned layer [40,41] and can be ascribed to a change in the magnetic order at the interface between IrMn and CoFeB, due to Mn interdiffusion and/or change in the AFM and FM grain size. This behavior is observed for all the Ru concentrations.

For comparison, we performed the measurement of H_E_ as a function of temperature on Co_40_Fe_40_B_20_(5 nm)/Ru(0.2 nm)/Ir_22_Mn_78_(6.5 nm)/Ru(2 nm) (Sample A5), where a Ru interlayer is placed between the AFM/FM interface, and no Ru inclusion is present in the IrMn layer. The result is shown in Figure 2a (green line, diamonds). In this case, the presence of the Ru interlayer significantly reduces the exchange bias field to 50 Oe, but does not allow tuning T_B_, which is comparable to that of sample A0. This result can be ascribed to the weaker exchange coupling between CoFeB and IrMn, due to the presence of a spacer between the FM and AFM interface [22].

It is worth noting that in all the cases, the blocking temperature is well below the critical temperature of layer intermixing [42] so that after a field cooling the exchange bias is always restored. Moreover, as will be discussed in Section 3.2, field coolings after annealing at higher temperature (290 °C) still give rise to similar values of exchange bias, indicating that the interdiffusion of Ru is limited and the system is stable.

As shown in Figure 3, the bottom-pinned samples show the same dependence of the blocking temperature and the exchange bias field on the Ru concentration as the top-pinned samples. Moreover, within the uncertainty of the measure, T_B_ is the same for equal Ru concentrations. We can conclude therefore that the control of the exchange bias strength via Ru doping can be applied both to the top-pinned and bottom–pinned configurations. Finally, it is worth to notice that, contrarily to the top-pinned samples, in the bottom-pinned case, the exchange bias field increases upon the first annealing, reaching values higher than in the annealed top-pinned configuration, given the same Ru concentration. This effect has already been observed in similar structures [40,41] and generally is ascribed to an improvement of the <111> IrMn texture [43]. An exception is represented by the 24% Ru sample, where the first annealing reduces the exchange bias also in bottom-pinned samples, probably due to the major role of interdiffusion at such high Ru concentrations.

In the as grown samples, higher values of exchange bias in top-pinned structures suggest a better initial <111> texture of IrMn with respect to the bottom-pinned case [41]. However, the top-pinned configuration is more unstable than the bottom-pinned one, as revealed by the decrease of H_E_ upon the first annealing shown in Figure 2b. This effect makes the exchange-bias value after annealing usually larger in bottom-pinned structures with respect to top-pinned ones. The marked difference between the top pinned and bottom pinned case can be ascribed to the inherently different growth mode of IrMn on CoFeB with respect to CoFeB on IrMn, leading to a different stability of the interfaces.

### 3.2. Magnetic Tunneling Junction with Two Pinned Electrodes

**Figure 4:**
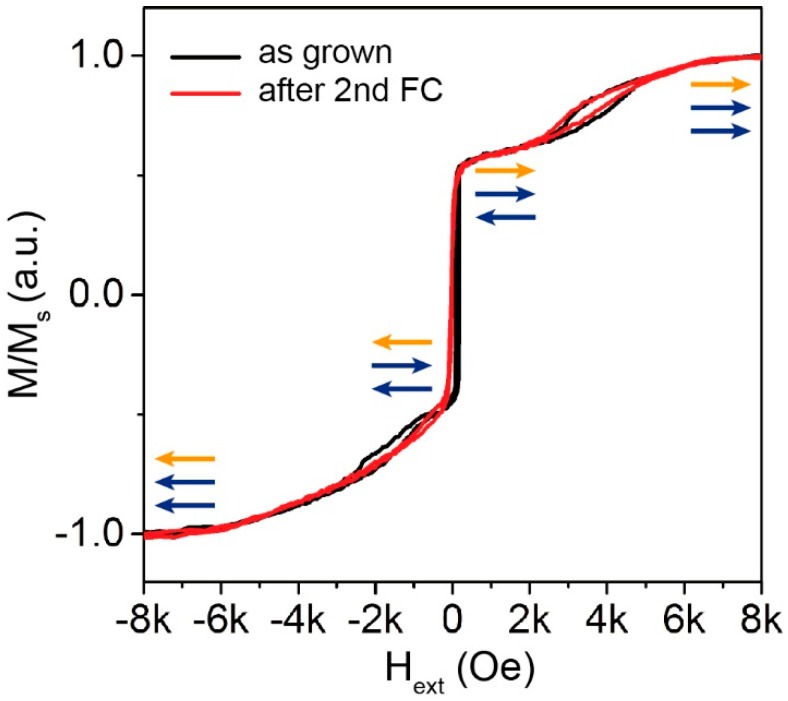
Magnetization as a function of the external magnetic field measured along the easy-axis of the bottom pinned layer on an as-grown sample (black line) and after the second field cooling (red line).

**Figure 5:**
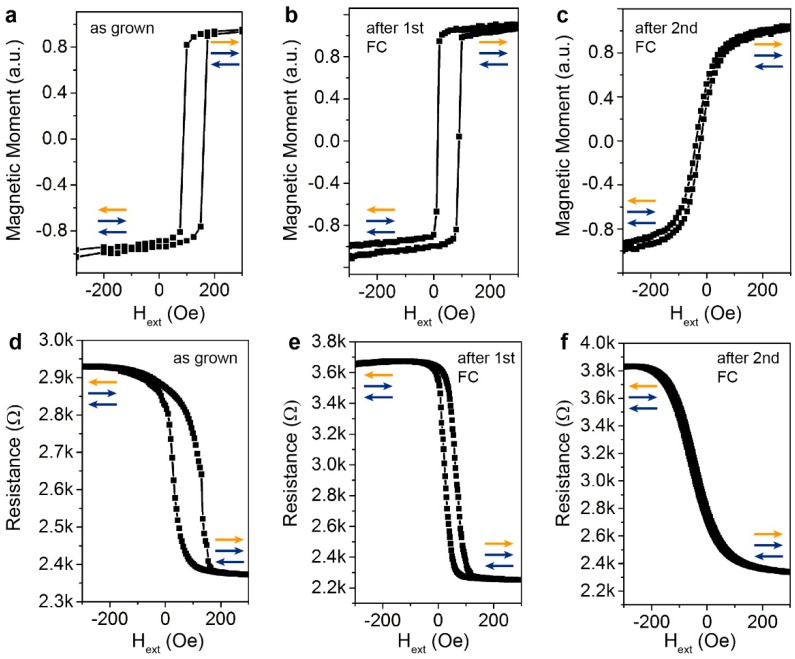
Upper panels: M-H loops measured along the easy-axis of the bottom pinned layer of the sensor (**a**) as grown; (**b**) after the first field cooling and (**c**) after the second field cooling. Lower panels: TMR curves of the sensor (**d**) as grown; (**e**) after the first field cooling and (**f**) after the second field cooling.

Figure 4 shows the M vs. H curve measured along the easy axis of the bottom electrode (the same direction of H_G_) on the non-patterned MTJ stack as grown (black line) and after the second field cooling (red line). Both measurements show the independent switching of the top-pinned CoFeB and of the bottom synthetic antiferromagnet (SAF) structure. The latter presents the typical spin flop configuration [14,22,44] during the rotation of the magnetization, giving rise to the lateral minor loops. The asymmetry of the loops is due to the presence of the IrMn antiferromagnetic layer exchange coupled to the CoFe film, which results in a shift of the right loop towards high positive fields. From the comparison of the two curves, one can observe that while in the as grown sample the exchange bias has the same direction in both the top and bottom CoFeB layer, after the second annealing, the central loop corresponding to the top CoFeB layer becomes a hard-axis loop (see Figure 5), due to the change of unidirectional anisotropy axis. It is worth noting that the second field cooling does not affect the switching of the SAF structure.

The top panels (a–c) of Figure 5 show the M vs. H hysteresis loops measured in the same direction as in Figure 4 for the top CoFeB layer after the different field cooling processes. The bottom panels (d–f) report the corresponding MR measurements with the magnetic field applied in the same direction. In the as grown sample (panels a,d), the top-pinned CoFeB shows an exchange bias field of about 130 Oe, which is reduced after the first annealing to about 50 Oe (panels b,e). This is consistent with what observed for sample A2 (see Figure 2), apart from minor discrepancies in the exchange bias field values, probably due to the different underlayers and thus texturing conditions, and to the interaction with the other magnetic films. This result indicates that the top-pinned configuration is stable until 290 °C with a limited Ru diffusion at interface. After the second field cooling, performed from T_FC2_ = 150 °C with H_FC2_ perpendicular to H_G_, the unidirectional anisotropy of the top CoFeB film is rotated by 90°, giving rise to the hard-axis hysteresis loop of Figure 5c,f.

Concerning the effect of the thermal annealing on the magnetoresistive curve, the MR is 23% in the as grown sample (panel d) and reaches 63% after the first annealing (panel e). The increment in the TMR is mainly due to the crystallization of the CoFeB electrodes and of the MgO barrier. No change in the MR value is observed after the second field cooling due to the much lower annealing temperature. As expected, after the second field cooling, the rotation of the anisotropy of the top CoFeB layer guarantees the linearity of the transfer curve of the MTJ, which features a sensitivity of about 3.6%/mT.

## 4. Discussion

In this paper, we show how the blocking temperature and the exchange bias field can be tuned by inserting controlled defects in the IrMn AFM layer. This tuning is crucial for engineering the transfer curve of magnetoresistive sensors, since it allows to set crossed anisotropies in the electrodes. Different concentrations of Ru can change the blocking temperature of the system from 220 °C to about 100 °C, preserving relatively high exchange bias field values. The combination of these two features, i.e., reduced blocking temperature and sizable exchange bias fields, can be ascribed to the high quality of the AFM/FM interface. The tuning of the blocking temperature by inserting Ru in the AFM layer is effective both in the top- and bottom-pinned structures, so that it can be easily applied in spin-valve and MTJ structures either with top or bottom detection layer.

In order to test our strategy in real devices, we fabricated MTJs with two pinned electrodes. In these devices, the top electrode is pinned with a 6.5 nm IrMn layer with a 17.5% concentration of Ru impurities. This composition gives rise to a 70 Oe exchange bias field combined with a blocking temperature lower than 150 °C, low enough to ensure that the second field cooling does not affect the bottom SAF structure. It is noteworthy that even with this Ru concentration and after annealing at 290 °C, no sizable degradation of the exchange bias is observed. The increase of TMR after the first annealing with respect to the as grown sample reveals that Mn interdifussion is limited to the interface between CoFeB and IrMn, while the interface with MgO is not compromised. For this reason, a thickness of 5 nm of top CoFeB layer has been chosen, but also with 3 nm thick CoFeB the same conclusion holds true (data not shown). Furthermore, we showed that the second field cooling performed at lower temperature does not affect the underlying SAF structure and, in particular, it does not rotate its unidirectional anisotropy axis. Indeed, the TMR value remains the same and MR measurements performed along other directions (data not shown) reveal that the maximum of the TMR is along the nominal easy-axis of the bottom layer, i.e., along H_G_.

Note that the low blocking temperature of the sensing electrode can in principle limit the temperature operating range of the sensor, but this could be extended towards the 200 °C range by using other AFM materials (e.g., PtMn) and/or by finely tuning the parameters of the second field cooling.

## 5. Conclusions

In this paper, we show that by including controlled concentrations of non-magnetic impurities in the AFM, a straightforward control on the blocking temperature of both top-pinned and bottom-pinned exchange bias systems can be achieved, preserving both high interfacial thermal stability and sizable exchange bias. This would represent an interesting strategy and an additional degree of freedom in the design of magnetic sensors with finely tailored response curve. Our approach can be applied to different AFM/FM systems, growth techniques and conditions. Furthermore, it can be combined with other tuning strategies, such as the insertion of a non-magnetic thin layer between the AFM and FM layers for further tuning the exchange bias strength. These results pave the way towards the development of ultrasensitive magnetic sensors, with the possibility to finely control the thermal stability, anisotropy strength and direction of the ferromagnetic electrodes.

## Figures and Tables

**Table 1:** Sputtering deposition parameters.

Material	Pressure (mTorr)	Power on 2 Inches Target (W)
Ta	3	100 DC
Ru	3	50 DC
Co_40_Fe_40_B_20_	3	58 DC
Ir_22_Mn_78_	3	50 DC
MgO	2	220 RF
Co_60_Fe_40_	12	200 RF

## Abbreviations

The following abbreviations are used in this manuscript:

SV	Spin Valve
MTJ	Magnetic Tunneling Junctions
SAF	Synthetic AntiFerromagnet
PMA	Perpendicular Magnetic Anisotropy
AFM	Antiferromagnet
TMR	Tunneling MagnetoResistance
SEM	Scanning Electron Microscope
VSM	Vibrating Sample Magnetometer
DC	Direct Current
FM	Ferromagnet
MR	MagnetoResistance
